# Modulation of corticospinal output in agonist and antagonist proximal arm muscles during motor preparation

**DOI:** 10.1371/journal.pone.0188801

**Published:** 2017-11-29

**Authors:** Cécilia Neige, Hugo Massé-Alarie, Martin Gagné, Laurent J. Bouyer, Catherine Mercier

**Affiliations:** 1 Center for Interdisciplinary Research in Rehabilitation and Social Integration, Québec, QC, Canada; 2 Department of Rehabilitation, Laval University, Québec, QC, Canada; University of Ottawa, CANADA

## Abstract

Previous studies have shown modulation of corticospinal output of the agonist muscle when a known-movement is prepared but withheld until a response signal appearance, reflecting motor preparation processes. However, modulation in the antagonist muscles has not been described, despite the fact that reaching movements require precise coordination between the activation of agonist and antagonist muscles. In this study, participants performed an instructed-delay reaction time (RT) task, with randomized elbow flexion and extension movements. The aim was to assess the time course modulation of corticospinal output in two antagonist muscles, by simultaneously quantified the amplitude of motor evoked potentials (MEPs) in biceps brachii and triceps brachii, and the amplitude and direction of elbow movements evoked by transcranial magnetic stimulation (TMS). Depending on the prepared movement direction, a specific modulation of corticospinal output was observed, MEPs and TMS-evoked movements amplitude being relatively greater for extension compared to flexion. At the end of motor preparation, a decrease in MEPs amplitude was observed for both biceps brachii and triceps brachii, regardless of the prepared movement direction. In contrast, the probability of evoking movement in the flexion direction and the amplitude of TMS-evoked movement decreased at the end of preparation for flexion, but not for extension. Together, these results confirm the existence of inhibitory processes at the end of the motor preparation, probably to avoid a premature motor response. Moreover, they provide evidence of differences in the corticospinal control of elbow flexor and extensor muscles with patterns of modulation that are not necessarily reciprocal during motor preparation.

## Introduction

Each of our voluntary movements requires a preparatory phase called action preparation, during which our brain elaborates motor commands in order to produce an optimal motor behavior (for a review see [1]). In a laboratory context, an instructed-delay reaction-time (RT) task is commonly used to assess action preparation [2,3]. In this task, two signals are presented successively to the participant. The first one, termed informative signal, can provide partial or full information concerning the forthcoming movement performed immediately after the appearance of the second signal termed response signal. The delay between the informative signal and the response signal corresponds to the motor preparation period, whereas the delay between the response signal and the onset of the electromyographic (EMG) activity in the agonist muscle corresponds to the movement initiation period. Studies using single-pulse transcranial magnetic stimulation (TMS) applied over primary motor cortex (M1) during different versions of the instructed-delay RT task allowed to assess mechanisms underlying action preparation by quantifying changes in the amplitude of motor evoked potentials (MEPs) (for reviews, see [4,5]).

Literature specifically targeting the motor preparation period during instructed-delay RT tasks has mostly reported a decrease in the agonist muscle corticospinal excitability [6–12]. This inhibition has been shown to be maximal when TMS was applied closer to the response signal appearance [9] and has been interpreted to reflect the prevention of a premature response initiation before the response signal appearance (i.e. false start) [13].

Together, these studies have focused on MEPs recording from the agonist muscle, in tasks only involving distal musculature (i.e. wrist or fingers). However, reaching movements, especially rapid ones, require precise coordination between the activation of agonist and antagonist muscles [14,15]. To the best of our knowledge, the simultaneous modulation of corticospinal output in agonist and antagonist muscles during the motor preparation period has never been explored. The purpose of the present study was therefore to investigate the modulation of corticospinal output during the motor preparation of elbow movements (flexion or extension). Importantly, corticospinal output was monitored by simultaneously looking at MEPs of two agonist/antagonist muscles acting at the elbow, biceps brachii (BB) and triceps brachii (TB), as well as at the amplitude and direction of elbow movements evoked by TMS at different time points during the motor preparation period. It was hypothesized that each muscle would show a reciprocal pattern of modulation depending on whether it is acting as an agonist vs. an antagonist. Moreover, it was expected that TMS-evoked movements would be more sensitive to the modulation of corticospinal excitability occurring during motor preparation than MEPs recording. This was expected because movement output results from the net interaction of all muscle contributions, contrary to MEPs which are muscle-specific. As such, TMS-evoked movements have the advantage of reflecting the net balance between the activation of several muscles that participate to the corticospinal output.

## Materials and methods

### Participants

Twenty-one healthy volunteers (7 women, 14 men; 25.7 ± 4.15 years old) participated in the study. All participants were right handed as assessed by the Edinburgh Handedness Inventory [16]. None of the participants reported neurological disorders or contraindications for TMS. All were free of pain or musculoskeletal disorder at the upper limbs.

The experiment was approved by the Ethics Committee of the Centre Intégré Universitaire de Santé et de Services Sociaux de la Capitale-Nationale–Installation Institut de réadaptation en déficience physique de Québec (Project #2016–072) in accordance with the Declaration of Helsinki and all participants provided their written informed consent prior to participation.

### Experimental design and task

**Fig 1:**
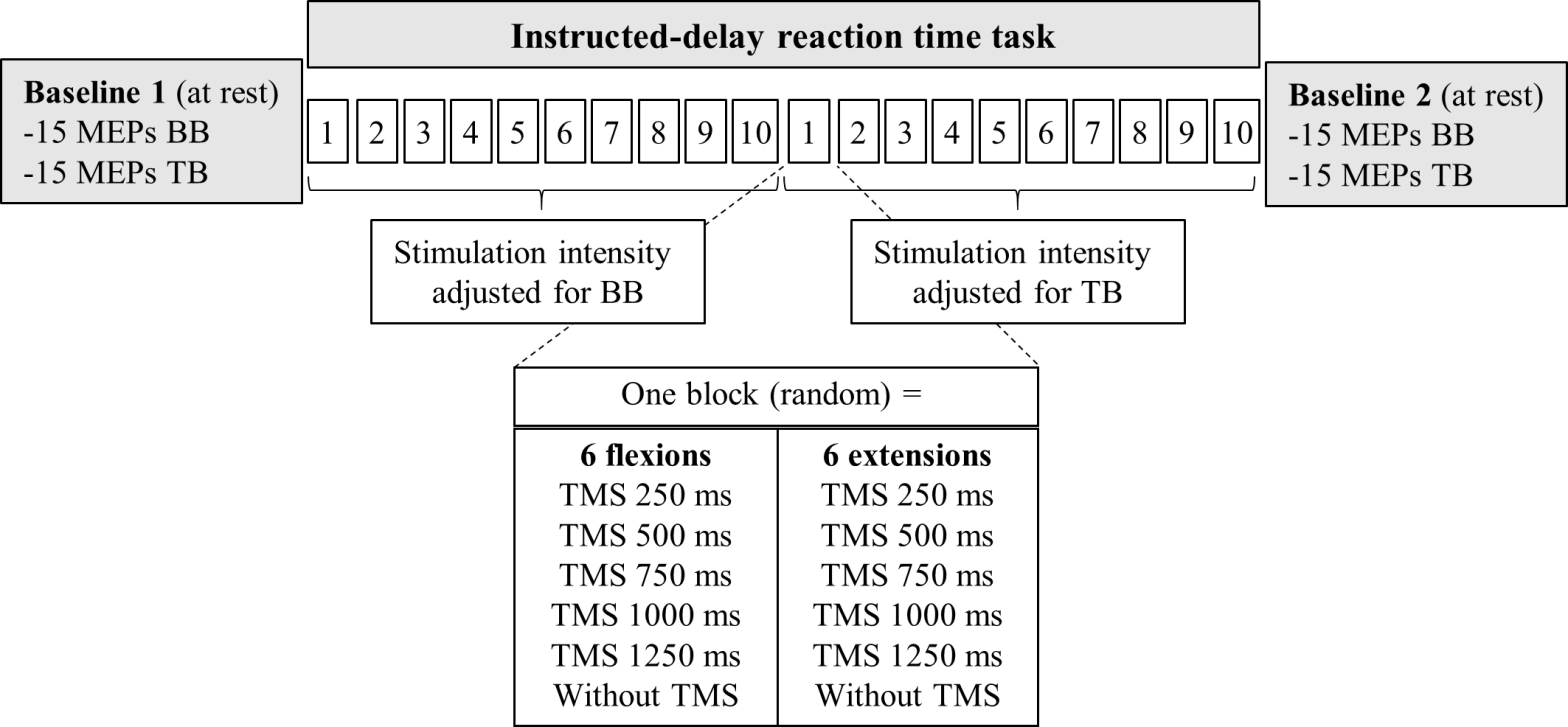
Experimental design. A first baseline (15 MEP for the biceps brachii and 15 MEP for the triceps brachii) was collected at rest. Then, participants realized the instructed-delay RT task composed of 10 blocks with a stimulated intensity adjusted for the biceps brachii then 10 others blocks were achieved and the intensity of stimulation was adjusted for the triceps brachii. One block consisted in five TMS-Intervals with one pulse per trial applied at a variable interval during the preparation phase (250, 500, 750, 1000 or 1250 ms after the informative signal) and a catch-trial without TMS for the two movement Directions. Finally, a second baseline was collected at the end of the experimentation. BB = biceps brachii; TB = triceps brachii.

The experiment was performed on a single session and required approximately 2 hours. As illustrated in Fig 1, experimental design comprised a familiarization with the task followed by three main parts: 1- baseline TMS measures (Baseline 1); 2- the instructed-delay RT task; and 3- a second TMS baseline (Baseline 2). During the familiarization, participants were exposed to the instructed-delay RT task. They had to complete a practice block of 14 movements (7 for each direction) without TMS while the experimenter monitored that they conformed to task requirements. In both Baseline 1 and 2, a set of 15 MEPs were obtained from each recorded muscle. Muscles were kept at rest and TMS parameters were adjusted separately for each muscle. In the instructed-delay RT task, corticospinal excitability was assessed at different time points during the motor preparation period. Five TMS-Intervals equally spaced from 250 ms were applied between 0 ms (informative signal appearance) and 1500 ms (response signal appearance). This provides a clear time-course of corticospinal excitability modulation from the beginning to the end of the motor preparation period. Since TMS parameters were optimized for the BB and the TB, the instructed-delay RT task was divided into two sections where MEPs from only one muscle were recorded (the order between muscles was pseudorandomized across subjects). For six subjects, MEPs from both muscles were recorded simultaneously as their TMS parameters were identical. For each muscle, 10 blocks of 12 randomized trials were tested. One block consisted in testing 5 TMS-Intervals (250, 500, 750, 1000 and 1250 ms) during the motor preparation period interleaved with 1 catch-trial without TMS for the 2 movement Directions (flexion, extension), for a total of 12 trials per block. The order of each condition (Direction and TMS-Intervals) was fully randomized within each block. Overall, 10 MEPs and 10 TMS-evoked movements per condition were recorded.

Arm movements were performed within a KINARM system consisting in a robotized exoskeleton interfaced with a 2D virtual environment (BKIN technologies Ltd., Kingston, ON, Canada). The KINARM allows recording of arm movement kinematics and presentation of visual information such as the position of the index finger tip (when the participant does not see his arm) and the position of different targets. Participant’s right arm was supported by the exoskeleton during the experimentation. Each movement was initiated from a Starting position corresponding to the position of the index finger tip when the shoulder was abducted at 90°, the upper arm was at 30° relative to the frontal plane and the elbow was flexed at 90°. Movements consisted in monoarticular elbow flexion or extension. A force field applied to the shoulder joint by the KINARM prevented any shoulder movement.

**Fig 2:**
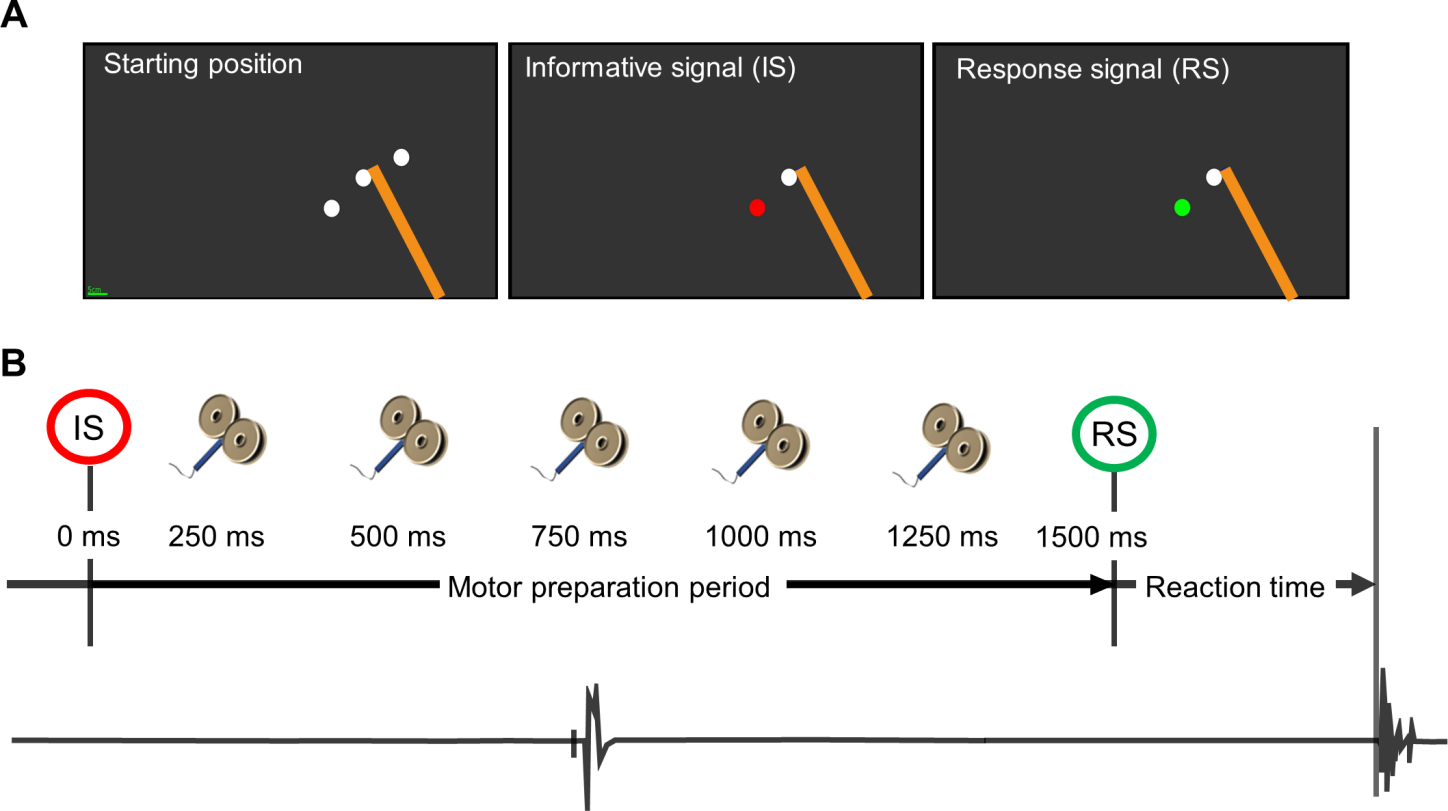
Schematic representations of the instructed-delay reaction time task. (A) Representation of the different targets used in the instructed-delay RT task and displayed on the screen of the KINARM device. This trial corresponds to a flexion movement. The orange bar represents the participant's right forearm (not visible to the subject). (B) Representation of the TMS-intervals (250, 500, 750, 1000 or 1250 ms; one stimulation per trial in random order) tested during the motor preparation period with an example of an electromyographic (EMG) trace of the biceps brachii during a stimulation applied at 750 ms. EMG onset in the agonist muscle was used to define the Reaction time.

Fig 2 illustrates the instructed-delay RT task. At the beginning of each trial, the robot passively moved the arm in the Starting position indicated by a white circle (radius = 1 cm). After 500 ms, two white targets (radius = 2 cm) were simultaneously presented and positioned at a distance equivalent to 15° of elbow flexion and extension respectively. After 1000 ms, only one target remained and turned red (Informative Signal) to inform the participant about the direction of movement to be performed. After a fixed motor preparation period of 1500 ms, the target turned green. This was the signal (Response Signal) to reach and pass through the target as quickly as possible without stopping in it, since the robot produced a dampening force field to break the movement after the target was reached.

#### EMG and kinematics recordings

EMG activity was recorded from pairs of surface Ag/AgCl electrodes (Kendall Medi-trace 200, Covidien) placed in bipolar configuration over the middle portion of the right BB and lateral head of the right TB. The ground electrode was positioned over the right acromion. EMG signals were amplified (X1000), band-pass filtered (10–500 Hz), digitized at a sampling rate of 1000 Hz by the KINARM data acquisition card (National Instruments PCI-6229 DAQ card, Austin, TX, USA) and stored on a computer for offline analysis. To minimize the risk of 60 Hz contamination of the EMG measurement arising from the KINARM robot or the virtual reality display, EMG signals were submitted, offline, to a second-order Butterworth notch filter (bandstop of 58 and 62 Hz). During the experiment, the EMG background was monitored by the experimenter to ensure a complete muscle relaxation at the beginning of each trial. Arm kinematics recorded form the KINARM motor encoders were recorded at 1000 Hz.

#### Transcranial magnetic stimulation

Single-pulse stimulation was delivered over the left primary motor cortex (contralateral to the dominant right arm) with a 70-mm figure-of-eight coil connected to a monophasic Magstim BiStim^2^ stimulator (The Magstim Co., Whitland, UK). The coil was placed tangentially to the scalp with the handle pointing toward the back and laterally at 45° away from the midsagittal line, resulting in a posterior-anterior current flow. Coil orientation and position was guided throughout the experiment by a neuronavigation system (Brainsight, Rogue research, Montreal, QC, Canada).

Because MEP amplitude evoked in both BB and TB can vary according to arm position [17–19], stimulation parameters were always defined with the participant’s arm in the KINARM at the Starting position. The optimal stimulation site on the scalp (hotspot) was defined as the location eliciting the largest MEP amplitude in both BB and TB. The resting motor threshold (rMT) corresponded to the lowest stimulation intensity required to evoke at least 5 out of 10 MEP of 50 μV [20]. The rMT was determined separately for each muscle. During the experiment, TMS stimulation intensity was fixed at 120% rMT of the tested muscle (or 130% if MEP were not reliably evoked at 120% during baseline measurements (n = 4)). It has to be noted that only BB data from 18 participants (6 women, 12 men; 25.4 ± 3.6 years old) and among them TB data from 12 participants (3 women, 9 men; 25.5 ± 2.8 years old) were used in the final analysis because it was not possible to evoke any MEP even at high stimulation intensity (>70% of maximal stimulator output (MSO)) in other recruited participants.

## Data analysis

All data analyses were performed using custom-made Matlab scripts (Mathworks inc., Massachusetts).

### Behavioral data

The reaction time was calculated for each condition from the time difference between the appearance of the Response Signal and the onset of the EMG burst in the agonist muscle (Fig 2B). The EMG burst onset was automatically determined based on a threshold method [21]. Mean baseline EMG and its standard deviation (SD) were calculated on a 249 ms window starting when the two white targets were presented to the participant. EMG onset was automatically detected after the Response Signal and corresponded to the moment when 75% of EMG points exceeded 3 SD over a period of 50 ms. Nevertheless, each trial was visually inspected and EMG burst onset was adjusted if necessary. When EMG burst was initiated less than 100 ms after the Response signal appearance, it was considered as a false start and the trial was removed from further analyses (3.3% of all trials, without difference between conditions).

Only trials without TMS were included for behavioral analyses based on previous studies that have shown that TMS stimulation applied during motor preparation period may impact RT [22,23].

Background EMG (root mean square, RMS) was monitored on the 100 ms preceding every TMS pulse to ensure that EMG during the instructed-delay RT task was comparable across conditions.

### Neurophysiological data

#### Motor evoked potentials (MEPs)

In order to quantify corticospinal excitability, the MEPs peak-to-peak amplitude was measured during Baseline conditions and during the instructed-delay RT task for each Direction, each TMS-Interval and each muscle tested.

#### TMS-evoked movements

Arm kinematics data necessary to determine the amplitude and direction of TMS-evoked movement were obtained from the KINARM motor encoders and sampled at 1000 Hz. Peak velocity of the fingertip displacement in the horizontal plane was first identified in a 150 ms window after TMS application to preclude any voluntary movement contamination. When different stimulation intensities were used for the BB and the TB because of a difference in rMT, the trials performed with the highest intensity of stimulation were used to obtained larger movements. Movement direction and amplitude were determined as the difference in elbow angle (in degrees) between the position at the moment of TMS application and the position reached at the peak velocity of the TMS-evoked movement within 150 ms following TMS. Therefore, positive values correspond to a TMS-evoked movement in the extension direction whereas negative values correspond to a TMS-evoked movement in the flexion direction.

As a reminder, the main objective of the study was to describe the specific modulation of corticospinal output during the motor preparation depending on the prepared direction and the timing of stimulation. Therefore, MEPs amplitude obtained for each trial were transformed separately for BB and TB into z-scores based on the mean and standard deviation of all motor preparation trials (regardless of the condition) and for each participant rather than test whether motor preparation differed from baseline taking at rest. Z-scores were then averaged for each Direction and TMS-Intervals. Such transformation into z-scores has been already done in previous studies assessing modulation of corticospinal excitability during motor preparation [6,24,25], and is particularly relevant when testing proximal muscle because of the inter-subject variability in MEP amplitude.

In order to probe changes in the direction of the TMS-evoked movement, the proportion (in %) of TMS-evoked movements in the flexion direction across all trials was calculated for each Direction and TMS-Interval. The amplitude of TMS-evoked movements was also normalised into z-scores, and averaged for each condition, similarly to the MEPs amplitude analysis.

## Statistical analysis

Normal distribution of the data was tested with the Shapiro-Wilk test, homogeneity of variances was assessed by Mauchly’s test and if sphericity assumption was violated, Huynh-feldt correction was applied.

Analyses of variance (ANOVAs) were performed on z-scores for MEPs amplitude independently for BB and TB, with Direction (flexion, extension) and TMS-Intervals (250, 500, 750, 1000 and 1250 ms) as within-subject factors. Similar ANOVAs were performed on the proportion TMS-evoked movements in the direction of flexion, as well as on z-scores for amplitude of TMS-evoked movements.

Additional analyses were performed to control for potential methodological biases. For each muscle, the RMS values were first compared between Baseline condition and instructed-delay RT task and then across all task conditions, using the same analyses described above for MEPs. In addition, a two-tailed paired sample *t*-test was used to compare raw MEP amplitude between Baseline 1 and Baseline 2 in order to ensure that there was no significant global changing in corticospinal excitability after the completion of the instructed-delay RT task. Another two-tailed paired sample *t*-test was used to compare RT across directions in order to describe behavioral performance in the motor preparation task. Statistical analyses were performed using SPSS 24 software (SPSS Inc., Chicago, IL, USA). Post-hoc analyses were performed using a Bonferroni correction for multiple comparisons. Uncorrected degrees of freedom and corrected *p* values for multiple comparisons are reported in the Results section. The α level for all analyses was fixed at .05. Partial eta squared (η_p_^2^) values are reported to provide the proportion of the total variance that is attributable to the tested factor or interaction [26]. Values in parentheses and data illustrated in figures represent mean ± standard error (SE).

## Results

### Methodological considerations

The analysis of pre-pulse background EMG level yielded no significant difference between Baseline conditions and Instructed-delayed RT task for any muscle (all p>.05). Moreover, concerning the mean RMS values, no difference across conditions of the instructed-delay RT task was observed for BB nor for TB (all p>.05). Therefore, any changes in MEP amplitudes cannot be attributed to differences in EMG level prior to the TMS pulse.

Mean MEP amplitudes did not significantly differed between the Baseline condition at the beginning vs. the end of the experiment (for BB: *Baseline 1* (0.31 ± 0.02 mV) *Baseline 2* (0.38 ± 0.03 mV) [*t*(_17_) = -1.5, p = .15]; for TB: *Baseline 1* (0.11 ± 0.01 mV), *Baseline 2* (0.12 ± 0.01 mV) [*t*(_11_) = -.47, p = .65]), indicating that corticospinal excitability in BB and TB did not change after the completion of the instructed-delay RT task.

At the behavioral level, reaction times did not differ according to the direction of movement (*Flexion* 317.8 ± 20.8 ms, *Extension* 301.9 ± 17.7 ms, [*t*(_17_) = -1.5, p = .15]).

### Modulation of MEPs in BB and TB during motor preparation

**Fig 3:**
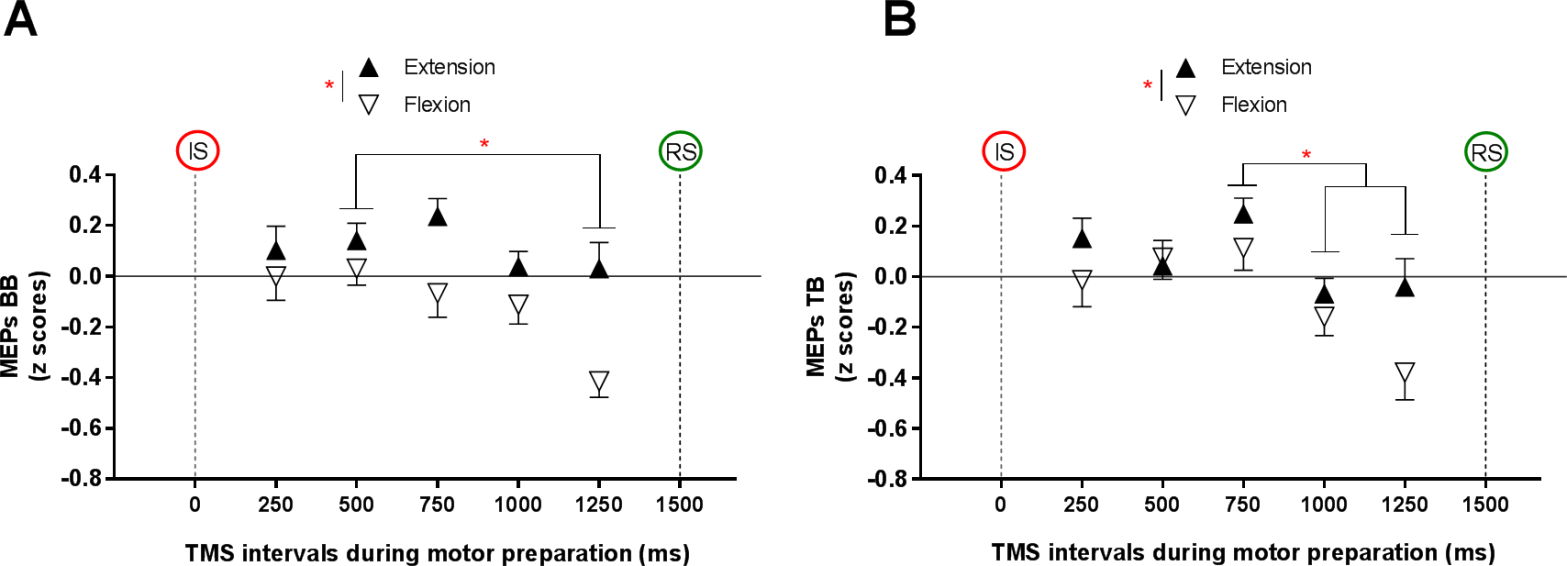
Mean (± standard error) z-scores for motor evoked potentials. (A) Comparison of motor evoked potentials (MEPs) recorded in biceps brachii during motor preparation period across the Direction and TMS-Intervals (n = 18). (B) Comparison of motor evoked potentials evoked (MEPs) in triceps brachii during motor preparation period across the Direction and TMS-Intervals (n = 12). The red asterisks indicate the statistically significant differences between conditions. BB = biceps brachii; TB = triceps brachii; MEPs: motor evoked potentials; TMS: transcranial magnetic stimulation; IS: informative signal; RS: response signal.

Fig 3 illustrates the time course of MEPs modulation for BB (Fig 3A) and TB (Fig 3B) during the motor preparation period according to movement Direction. The ANOVA revealed a main effect of Direction for both BB (F_(1,17)_ = 17.7, p = .001, ƞ_p_^2^ = .51) and TB (F_(1,11)_ = 6.1, p = .03, ƞ_p_^2^ = .36), showing that mean MEPs amplitude was relatively greater during the preparation of extension compared to flexion in both muscles. Importantly this effect seems to be driven mainly by a decrease in MEPs amplitude during flexion preparation, although the interaction between TMS-Intervals and Direction was not significant for either BB (F_(4,68)_ = 1.9, p = .12) or TB (F_(4,44)_ = 1, p = .39).

A significant main effect was also observed for TMS-Intervals in both BB (F_(4,68)_ = 3.2, p = .02, ƞ_p_^2^ = .16) and TB (F_(1,11)_ = 5.1, p = .002, ƞ_p_^2^ = .32). Post hoc analyses showed that for BB, corticospinal excitability was lower at the last TMS-Interval (i.e. just before the Response signal appearance: 1250 ms vs. 500 ms (p = .04)). For TB, post hoc analyses revealed that corticospinal excitability was similarly lower at 1000 ms and 1250 ms compared to 750 ms (p = .002 and p = .01, respectively).

### Modulation of TMS-evoked movements during motor preparation

#### Direction

**Fig 4:**
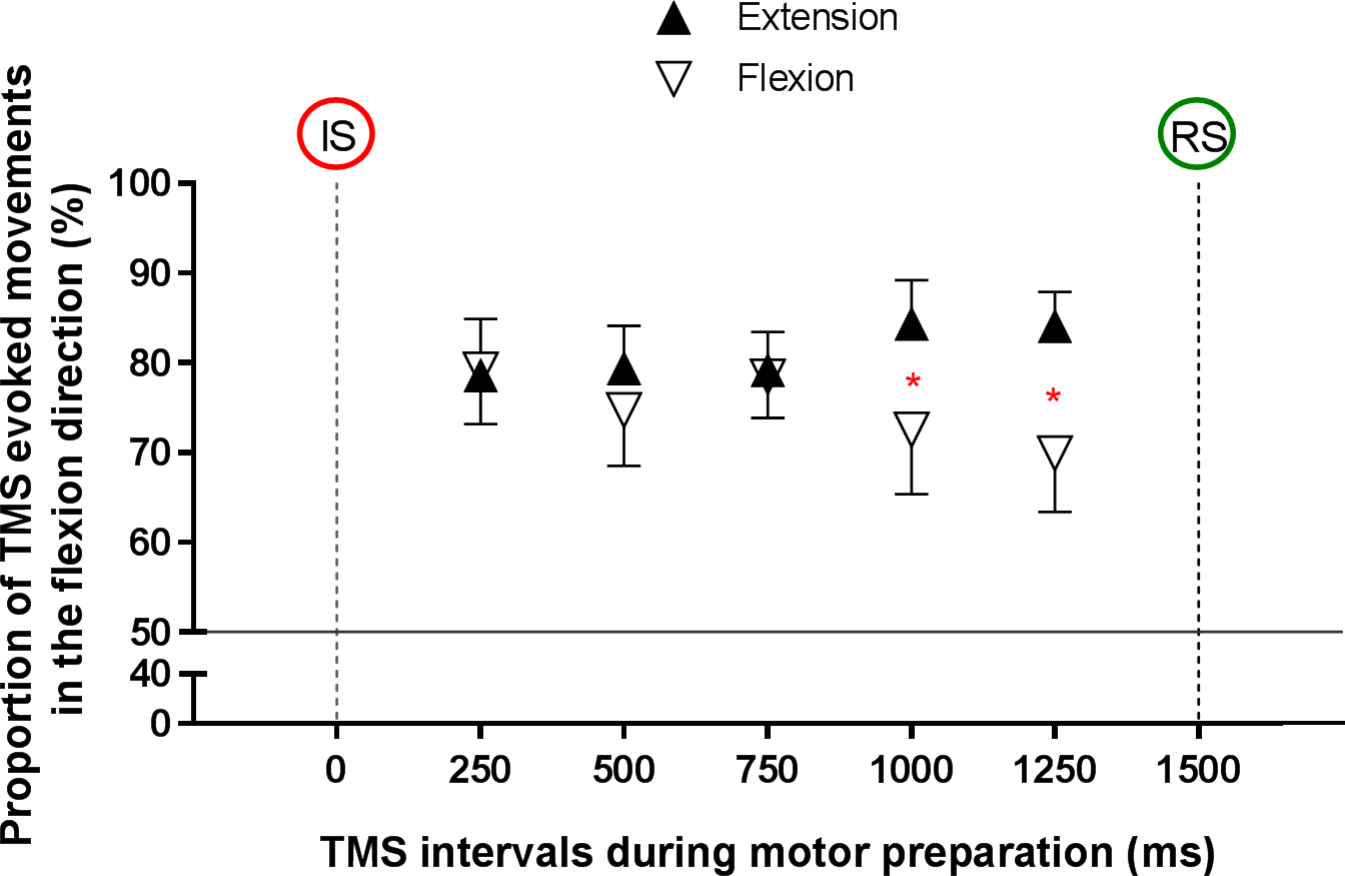
Mean (± standard error) proportion for TMS-evoked movements in the flexion direction during motor preparation period, depending on the Direction and TMS-Intervals. The red asterisks indicate the statistically significant differences between conditions. TMS: transcranial magnetic stimulation; IS: informative signal; RS: response signal.

Fig 4 shows the proportion of TMS-evoked movements in the flexion direction under each experimental condition.

Across all conditions, TMS-evoked movements were most of the time in the flexion direction (78 ± 23%). Statistical analysis indicated a trend for an effect of Direction (F_(1,17)_ = 3.8, p = .067) and no main effect of TMS-Intervals (F_(4,68)_ < 1, p = .89). However, a significant Direction X TMS-Intervals interaction was observed (F_(4,68)_ = 3, p = .03, ƞ_p_^2^ = .15). Post-hoc analysis revealed that the proportion of TMS-evoked movements in the flexion direction decreased when the participant prepared a flexion compared to an extension at TMS-Intervals 1000 ms (p = .047) and 1250 ms (p = .007).

#### Amplitude

**Fig 5:**
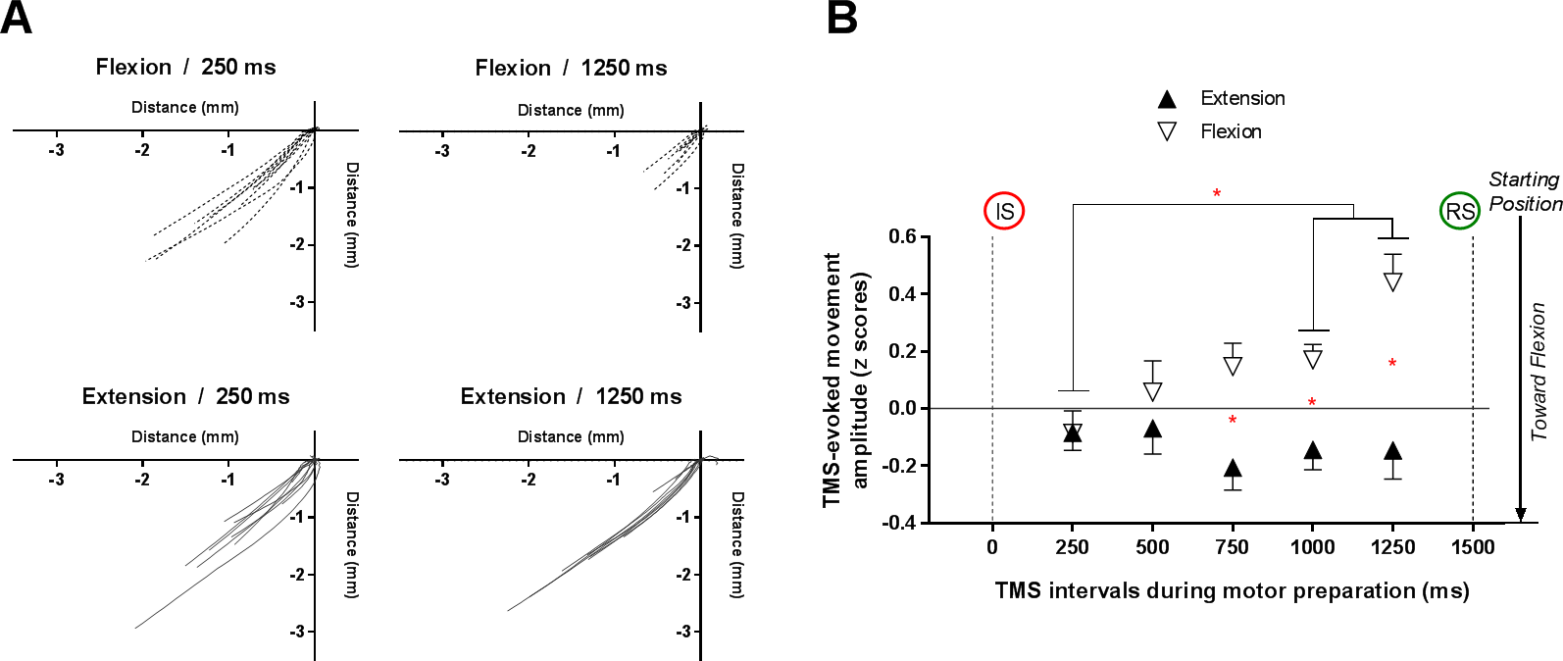
Mean (± standard error) z-scores for TMS-evoked movements amplitude. (A) Individual raw TMS-evoked movements during motor preparation period for one representative participant for Flexion (250 ms and 1250 ms) and Extension (250 ms and 1250 ms). Origin of each vector is centered at X = 0 and Y = 0 in order to permit comparison of direction and amplitude. (B) Comparison of changes in TMS-evoked movements during motor preparation period depending on the Direction and TMS-Intervals. Note that higher z-scores values correspond to smaller TMS-evoked movement amplitudes. The red asterisks indicate the statistically significant differences between conditions. TMS: transcranial magnetic stimulation; IS: informative signal; RS: response signal.

Fig 5 illustrates the normalised amplitude of TMS-evoked movements during the motor preparation period, according to Direction and TMS-Intervals. Representative vectors of TMS-evoked movements for one participant are shown in Fig 5A. Statistical analysis on normalised data revealed a clear main effect of Direction (F_(1,17)_ = 11.4, p = .004, ƞ_p_^2^ = .40). A trend for a main effect of TMS-Intervals was also observed (F_(4,68)_ = 2.2, p = .08). Finally, a significant Direction X TMS-Intervals interaction was observed (F_(4,68)_ = 3.8, p = .007, ƞ_p_^2^ = .18). Post-hoc analyses indicated that for flexion, TMS-evoked movements were smaller during the last two TMS-Intervals compared to the first TMS-Interval: 250 ms vs. 1000 ms (p = .039); 250 ms vs. 1250 ms (p = .002) whereas no modulation according to TMS-Intervals was observed for extension (all p>.05). Moreover, results indicated that TMS-evoked movements were smaller for flexion compared to extension during the three last TMS-Intervals before the Response signal appearance: 750 ms (p = .01); 1000 ms (p = .005) and 1250 ms (p = .001).

## Discussion

The main objective of this study was to investigate, for the first time, the simultaneous modulation of corticospinal output across time, in proximal agonist and antagonist arm muscles during motor preparation of elbow movements. The results revealed a specific modulation during the instructed-delay RT task according to the direction, MEPs and TMS-evoked movements amplitude being relatively higher during the preparation of extension compared to flexion, mainly due to a decrease in MEPs amplitude during flexion preparation. Indeed, at the end of motor preparation, a decrease in MEPs amplitude was observed for both BB and TB, regardless of movement direction. Again this main effect appears to be driven mainly by the decrease in MEPs amplitude during flexion preparation, although no significant interaction was found. In addition, both the proportion of TMS-evoked movements toward flexion and the amplitude of TMS-evoked movement decreased at the end of preparation for flexion movement but not for extension.

### Modulation of corticospinal excitability depending on the direction during motor preparation

Contrary to our hypothesis, a reciprocal pattern in MEPs for flexor (BB) or extensor (TB) muscles depending on whether they were acting as agonist vs. antagonist has not been observed. Our results have rather demonstrated a specific modulation of corticospinal excitability according to the Direction that is relatively similar between elbow flexor and extensor muscles. This suggests that the fact that a muscle will be involved as the agonist vs. the antagonist in the movement has no impact on the modulation of MEPs amplitude, i.e. that MEPs changes are not necessary directly related to upcoming changes in muscle activity. To our knowledge, only one study demonstrated that corticospinal excitability increased when BB will act as an agonist (prior to a BB contraction) and decreased when BB will act as an antagonist (prior to a pronator teres contraction) [27]. Importantly, this difference was observable only from 200 ms prior voluntary contractions that were paced on the regular beat of a metronome, a task quite different from the task used in our study. Previous studies also reported such a specific modulation during simple RT task. It has been reported a progressive increase in corticospinal excitability of the agonist muscle from ≈100 ms before the initial EMG burst [28–33] and a decrease in corticospinal excitability of the antagonist muscle from 60 ms before the EMG onset [10,29]. Thus, it is possible that a selective modulation of corticospinal excitability depending on the agonist or antagonist role of the muscle is only observable after the response signal (i.e. within the motor initiation period).

### Modulation of corticospinal excitability depending on the TMS-Interval during motor preparation

Patterns of modulation of MEPs from BB and TB during motor preparation are quite similar, that is, a decrease during the last TMS intervals before the response signal appearance. Interestingly, the same pattern of results was observed in a study investigating the modulation of the first dorsal interosseous muscle corticospinal excitability during motor preparation of an index movement [9]. In the latter study, no modulation was observed close to the informative signal appearance, whereas corticospinal excitability decreased at the end of motor preparation period. This inhibition is believed to reflect the “*impulse control*” mechanism acting to avoid a premature response before the response signal appearance (i.e. false start) [7]. This inhibitory process has been suggested to arrive from dorsal premotor cortex [34] projecting on M1 and on the spinal cord [35,36]. Although no significant interaction between the Direction and TMS-Intervals was found in the present study, this phenomenon was predominantly observed during flexion preparation.

### Modulation of corticospinal output depending on direction and TMS-Intervals during motor preparation

Results on MEPs for both muscles showed that corticospinal excitability is on average lower during preparation for flexion compared to extension regardless of TMS-Intervals, with a decrease of excitability just prior to the response signal regardless of the Direction. Results on both TMS-evoked movements direction and amplitude showed a clear direction-specific effect that suggests a net reduction in the flexors/extensors corticospinal excitability balance during the preparation for flexion, without such change during the preparation for extension movements. This suggests a greater need for a super-imposed inhibitory control during preparation of flexion movements, and such inhibitory control appears to be particularly needed when the response signal is imminent. The fact that the large majority of TMS-evoked movements were in the flexion direction indicate a stronger corticospinal output for flexor muscles compared to extensors (at least in the posture in which subjects were tested). Moreover, for the 12 participants who presented MEPs in the two tested muscles, resting motor threshold were on average higher in TB than BB (respectively 43.6 ± 6.1%MSO vs. 41.4 ± 5.1%MSO; p = .04). Moreover, mean MEPs amplitude obtained at 120% rMT during the task were systematically higher in BB compared to TB (respectively 0.77 ± 0.52 mV vs. 0.21 ± 0.10 mV; p < .0005), reflecting a steeper slope in the recruitment curve for BB. This asymmetry in cortical output between flexors and extensors could explain why a decrease in corticospinal output was observed during preparation for flexion movement only, as the need to avoid a premature response before the response signal appearance might be more prominent. Thus, this superimposed inhibitory control during preparation for flexion movements, especially when the response signal is imminent, is probably not necessary during preparation for extension movements and may explain the absence of modulation in corticospinal output. Differences in the control of the flexors and extensors of the forearm as well as corticospinal control of each muscle group has been previously shown in human and animal studies [37–39]. Interestingly, experiments in monkeys have revealed that intracortical motor cortex stimulation evokes inhibitory effects more often in flexor muscles than in extensors [40,41]. Moreover, it has been shown that MEPs amplitude evoked in the forearm flexor increased more than MEPs evoked in the forearm extensor during the motor initiation period [42]. Our results also suggest differences in the corticospinal control of elbow flexor and extensor muscles in humans during motor preparation period, with a greater corticospinal output for the flexor muscles. Overall, this suggests that the corticospinal system is primed for flexion movements, but that this bias is counterbalanced by inhibitory mechanisms specifically regulating these movements. Developing a better understanding of the normal neurophysiological control of upper limb flexor and extensor muscles is a pre-requisite to being able to explain the mechanisms underlying pathological motor behaviors, such as “abnormal synergies” observed after stroke [43].

### Methodological considerations

Proximal movements involve several muscles with most muscles comprising different compartments. As expected, TMS-evoked movements results provided an information that was complementary to the MEPs by revealing the net balance between the activation of several muscles (while MEPs are muscle-specific). This measure could be useful in future protocols to assess additional aspects of motor preparation for reaching movements in healthy and pathological populations [44,45].

Another methodological point in the present study is that our z-scores normalisation is slightly different than previous studies using baseline conditions during the inter-trial intervals or at the onset of a fixation cross preceding the informative signal appearance to normalise the MEPs amplitude [46,47]. This approach is often preferred to recording the baseline at rest to avoid any attentional effect known to take place during motor preparation on excitability. However, z-scores normalisation also provides specific modulation according to Direction and TMS-Intervals and allowed to account for attentional effects and for inter-individual differences in proximal corticospinal excitability.

A critical point in our study is that it was difficult to evoke MEPs in TB lateral head in our group of participants (only 12/18 showed a response) and their amplitude were quite small, even within the motor preparation period. However, the MEPs amplitudes obtained were comparable to those reported for TB in a previous study [19]. Mean amplitude of TMS-evoked movements were also small, but again comparable to that reported in a previous study showing that this measure is very consistent [48].

## Conclusion

Overall, this work provides evidence of differences in the corticospinal control of elbow flexor and extensor muscles with patterns of modulation that are not necessarily reciprocal during motor preparation. This study also provides further methodological evidence that corticospinal output in agonist/antagonist proximal muscles during elbow movement preparation can be probed by combining the use of TMS with that of a robotized exoskeleton [48].
